# Reliable measurements of extracellular vesicles by clinical flow cytometry

**DOI:** 10.1111/aji.13350

**Published:** 2020-10-08

**Authors:** Martine Kuiper, Arthur van de Nes, Rienk Nieuwland, Zoltan Varga, Edwin van der Pol

**Affiliations:** ^1^ Biomedical Engineering and Physics Amsterdam University Medical Centers, Location AMC University of Amsterdam Amsterdam The Netherlands; ^2^ Laboratory Experimental Clinical Chemistry Amsterdam University Medical Centers, Location AMC University of Amsterdam Amsterdam The Netherlands; ^3^ Vesicle Observation Center Amsterdam University Medical Centers, Location AMC University of Amsterdam Amsterdam The Netherlands; ^4^ Dutch Metrology Institute VSL Delft The Netherlands; ^5^ Biological Nanochemistry Research Group Institute of Materials and Environmental Chemistry Research Centre for Natural Sciences Budapest Hungary

**Keywords:** calibration, data interpretation, extracellular vesicles, flow cytometry, standardization

## Abstract

Extracellular vesicles (EVs) are cell‐derived particles with a phospholipid membrane present in all body fluids. Because EV properties change in health and disease, EVs have excellent potential to become biomarkers for diagnosis, prognosis, or monitoring of disease. The only technique capable of detecting, sizing, and phenotyping a million of EVs within minutes is (clinical) flow cytometry. A flow cytometer measures light scattering and fluorescence signals of single EVs. Although these signals contain valuable information about the presence and composition of EVs, the signals are expressed in arbitrary units, which make the comparison of measurement results impossible between instruments and laboratories. Additionally, unintended and undocumented variations in the source, preparation, and analysis of the sample lead to orders of magnitude variations in the measured EV concentrations. Here, we will explain the basics, challenges, and common misconceptions of EV flow cytometry. In addition, we provide an overview of recent standardization initiatives, which are a prerequisite for comparison of clinical data and thus for clinical biomarker exploration of EVs.

## 1 EXTRACELLULAR VESICLES

Extracellular vesicles (EVs) are cell‐derived particles with a phospholipid membrane that are present in all body fluids. Because EV properties may change in disease, EVs have excellent potential to become biomarkers for diagnosis, prognosis, and monitoring of diseases including cancer, cardiovascular diseases, and preeclampsia.^1^, ^2^, ^3^, ^4^ Before EVs can be used as biomarkers, however, reliable measurements are required. Reliable measurements of EVs are difficult because (a) most EVs are smaller than 200 nm,^5^ which is considerably smaller than cells, thereby making EVs difficult to detect, (b) most body fluids contain EVs from different cell types,^6^ and (c) most body fluids contain other particles than EVs within the size range of EVs.^7^ A technology able to detect EVs, differentiate EVs from different cell types, and distinguish EVs from non‐EV particles is flow cytometry.

## 2 FLOW CYTOMETRY

Flow cytometers (FCMs) are commonly available in clinical laboratories and can provide information about the concentration, phenotype via fluorescent labeling, refractive index (RI), and size of single EVs.^8^ To understand pitfalls and challenges that are specific to EV flow cytometry, we will first explain the basics of flow cytometry regarding fluidics, light scattering, and fluorescence, followed by a description of the standardization efforts ongoing in the EV field, and the prospects of EV research. This manuscript focuses on clinical flow cytometers and therefore does not cover cell sorters, imaging FCMs, or nanoparticle FCMs, which we define as FCMs specialized in the detection of EVs smaller than 100 nm.^9^ In contrast to clinical flow cytometers, nanoparticle FCMs (a) have fluidics and optics unsuitable for cell characterization, (b) accomplish high‐sensitivity at the expense of throughput and therefore have a maximum count rate <1000 events per second, and (c) are (still) rarely available.

### 2.1 Fludics

**FIGURE 1:**
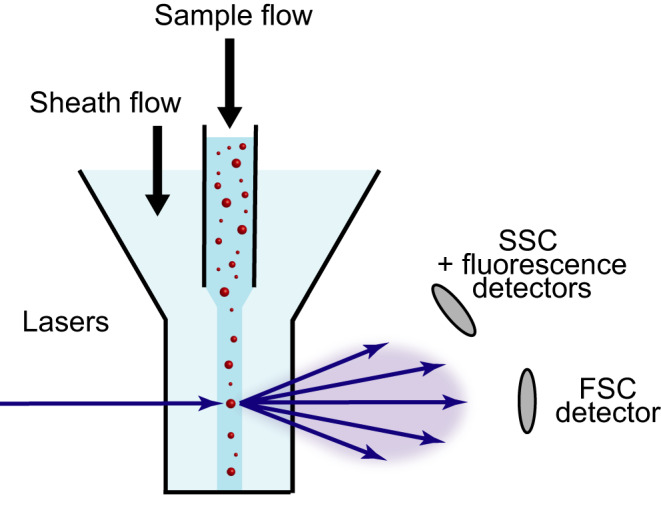
A flow cytometer utilizes a sheath flow to hydrodynamically focus the sample flow. Extracellular vesicles (EVs) in the sample pass through a focused laser beam and scatter light in all directions. A fraction of the scattered light is collected by lenses, which are typically placed in the forward scattering (FSC) and side scattering (SSC) direction. The fluorescence of EVs labeled with fluorophores is collected by the SSC lens and spectrally filtered to differentiate fluorescent signals from SSC

An FCM hydrodynamically focusses an EV sample into a stream of sheath fluid, as shown in Figure 1. Because the sheath fluid has a higher flow rate than the sample, the sample flow becomes relatively narrow, thereby centering the EVs in the flow cell. Increasing the sample flow rate increases the diameter of the sample flow and the number of EVs detected per unit time.^10^, ^11^, ^12^

Sample flow rates used to detect cells typically range between 10‐50 µL/min,^10^ which results in a sample flow diameter in the size range of cells. Hence, single cells will pass through the center of the focused laser beam. However, because most EVs are smaller than 200 nm, EVs are considerably smaller than the sample flow diameter used for cell detection, which can affect EV measurements in two ways. First, EVs may pass the focused laser beam at different locations, thereby leading to variation in the detected signals. Second, a relatively large sample flow diameter increases the probability that multiple EVs and other particles are simultaneously illuminated, which may lead to an unwanted effect called swarm detection (see section Common misinterpretations). To prevent swarm detection and to ensure that EVs are centered in the laser beam, EVs are often detected with the lowest flow rates present on FCMs, typically in the range of 3‐12 µL/min.^10^

The choice of sample dilution buffer and sheath fluid depends on the application.^10^ To prevent background scattering caused by RI differences between the interface of the sample and sheath fluid, the sample dilution buffer and sheath fluid are ideally the same. EVs need to be diluted in an isotonic buffer solution, such as phosphate‐buffered saline (PBS), to prevent damage to the EVs by osmosis. To match the RI, PBS therefore seems an obvious choice for the sheath buffer. However, PBS may increase the formation of salt crystals in the fluidics, which causes artefacts such as additional background noise, clogging, or loss of laminar flow, making especially dim EV signals unreliable.^10^, ^11^ Due to these artifacts, several new flow cytometers dedicated to EV detection use deionized water as a sheath fluid.^13^, ^14^ The optimal sample dilution buffer and sheath fluid are subject for ongoing research.

### 2.2 Light scattering

When an EV is illuminated by the laser beam, the EV scatters light into all directions. A typical FCM detects the scattered light in the forward scattering (FSC) and sideward scattering (SSC) directions, as shown in Figure 1.^12^ In the FSC direction, the laser beam is blocked by an obscuration bar to prevent laser light impinging upon the detector. Although scattered light contains valuable information about the EVs, such as the diameter, getting access to this information is not straightforward for two reasons. First, the light scattering intensity is measured in arbitrary units, which differ between FCMs, making data comparison difficult. Secondly, the signal is difficult to interpret, because the dependence of light scattering on EV properties is complicated.

#### 2.2.1 Parameters affecting light scattering detection

**FIGURE 2:**
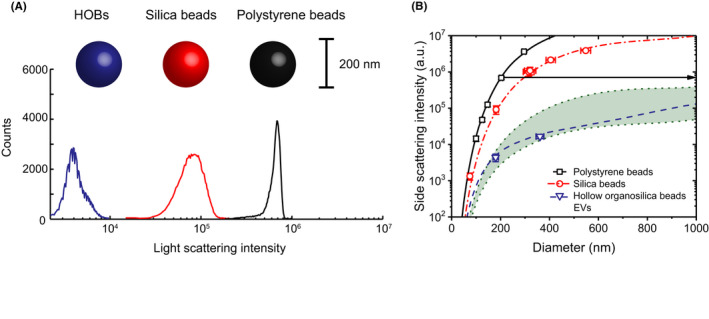
A, The scattering intensities of hollow organosilica beads (HOBs) with a diameter of 180 nm, silica beads with a diameter of 183 nm and polystyrene beads with a diameter of 203 nm show that beads with higher refractive indices have a higher light scattering intensity. Polystyrene and silica beads have a refractive index of 1.633 and 1.475, respectively. Because the core of HOBs contains water, HOBs scatter light less efficiently than both polystyrene and silica. B, Side scattering intensity versus diameter measured (symbols) by flow cytometry (A60‐Micro, Apogee Flow Systems) and calculated (lines) with Mie theory for polystyrene beads (squares), silica beads (circles), HOBs (triangles), and EVs (shaded area between dotted lines). Please note that the vertical scale is logarithmic. Mie theory calculations are performed with Rosetta Calibration software (Exometry, The Netherlands) assuming a refractive index of 1.633 for polystyrene beads and 1.475 for silica beads. HOBs are modeled as core‐shell particles, with a core refractive index of 1.343, a shell refractive index of 1.475, and a shell thickness of 10 nm. EVs were also modeled as core‐shell particles, but with a core refractive index ranging from 1.343 to 1.36, a shell refractive index of 1.46, and a shell thickness of 5 nm. The side scattering intensity increases with increasing diameter, but also with increasing refractive index. Hence, for this flow cytometer, polystyrene beads of 200 nm scatter light more efficiently than 1000 nm EVs (arrow)

The detected light scattering signal depends on both the EVs and the (clinical) FCM. Properties of EVs affecting light scattering are the diameter, RI, and shape. The RI depends on the EV composition and is a property that is often overlooked. To emphasize the importance of the RI, Figure 2A shows the measured (A60‐Micro, Apogee Flow Systems) scattering intensities of similar‐sized polystyrene (PS) beads, silica beads, and hollow organosilica beads (HOBs). At a typical illumination wavelength of 405 nm, the RI is 1.63 for PS beads, and 1.44‐1.47 for silica beads. HOBs have a ~10 nm thick shell with an RI equal to silica. Although the beads are similar in size, HOBs scatter 20‐fold less light than silica beads and 175‐fold less light than PS beads. To illustrate how both the RI and diameter affect light scattering, Figure 2B shows SSC vs diameter for the aforementioned beads and EVs. Generally, light scattering increases with increasing diameter and RI.^15^, ^16^, ^17^

Properties of the FCM that affect the detected light scattering signal involve the power and wavelength of the illumination and the collection angle of the lens. The scatter to diameter relation in Figure 2B therefore differs between FCMs. Because most EVs are spherical, light scattering detected by an FCM can be well‐described by Mie theory.^5^, ^17^, ^18^ Mie theory takes into account all the aforementioned parameters of both the FCM and EVs and is valid for all EV sizes.

#### 2.2.2 From arbitrary units to comparable units

To make results insightful and comparable, the arbitrary units of light scattering should be converted to SI units. Such a calibration is generally performed using well‐characterized reference beads, such that the data are physically accurately expressed and can be converted to SI units. Figure 2B shows how Mie theory can be used to relate the measured scatter signals to the diameter of EVs, thereby assuming an effective RI of EVs based on the measured RI of lipid bilayers (shell) and the cytosol (core) of cells.^17^ To implement sizing of EVs by flow cytometry in the workflow, different software are available to apply Mie theory, such as FCM_pass_ and Rosetta Calibration.^19^, ^20^ Daily runs of beads without applying Mie theory, as is common practice in the field, do not convert arbitrary units to SI units and are therefore not a calibration. Nevertheless, daily runs of beads are useful to set up the FCM and to check whether the instrument measures consistent over time and are thus a quality control.

#### 2.2.3 Common misinterpretations

Despite the availability of free and commercial software to size EVs with light scattering,^17^ the most common pitfall is the use of PS beads to define gates for EV detection. Figure 2B shows, for example, that SSC of a 200 nm PS bead corresponds to EVs larger than 1000 nm. Despite their lower RI, also silica beads scatter considerably more light than similar‐sized EVs. Moreover, the scatter to diameter relation depends on the collection angles, which differ between FCMs. Thus, neither PS nor silica beads can be used to gate EVs because (a) the RI of beads and EVs differ and (b) the collection angles differ between FCMs. Of the beads displayed in Figure 2B, only HOBs mimic the physical light scattering properties of EVs.^16^ Hence, only HOBs are suitable to set true EV size gates without correction with Mie theory. Table 1 summarizes whether the aforementioned reference beads and methods can be used as a calibration or quality control.

**TABLE 1:** Overview of approaches to standardize light scattering detected with flow cytometry over time

Time (years)	Approach	Example	Quality control	Calibration	EV size	Ref.
1988	Gate based on light scattering of activated platelets	EVs are smaller than platelets; thus, events with lower scattering than platelets are labeled “EVs”	No	No	Smaller than platelets	Sims,^51^ Nieuwland^52^
2004	Gate based on light scattering of PS beads	Megamix	Yes	No	No	Simak,^53^ Robert,^42^ Lacroix^43^
2011	Light scattering theory to calculate scatter to diameter relation, not taking into account collection angles	Sub‐micrometer PS beads and silica beads may gate EVs larger than 1 µm	Yes	No	No	Chandler^54^
2012	Light scattering theory to calculate scatter to diameter relation, taking into account collection angles	Use flow cytometry to make size distributions of EVs, like FCM_PASS_,^19^ Rosetta Calibration^20^	Yes	Yes	Yes	van der Pol,^15^, ^20^ Welsh,^19^ Tian^55^
2015	Gate based on light scattering of silica beads	ApogeeMix	Yes	No	No	Pospichalova^13^
2017	Gate based on different PS bead sizes for FSC and SSC signals	Megamix‐Plus FSC and Megamix‐Plus SSC	Yes	No	No	Cointe^44^
2018	Gate based on light scattering of HOBs, which have an RI distribution similar to EVs	Hollow organosilica beads	Yes	Yes, at two diameters	Yes, at two diameters	Varga^16^

Note

For each approach, the table provides an example, the suitability to function as quality control and calibration, the suitability to determine the EV size, and a literature reference.

Abbreviations: EVs, extracellular vesicles; FSC, forward scattering; PS, polystyrene; RI, refractive index; SSC, sideward scattering.

Swarm detection is a special case of coincidence detection, where instead of two or a few particles, multiple (tenths to hundreds) particles at or below the detection limit are simultaneously and continuously present in the laser beam of the flow cytometer and erroneously measured as single counts.^15^ Swarm detection can be prevented by diluting the sample prior to analysis, or by lowering the sample flow rate, which will result in higher sensitivity.^15^, ^21^ The presence of swarm detection can be checked by a dilution series of the EV sample. Swarm detection is present when (a) EV concentrations do not scale linearly and (b) the median scattering intensity increases with decreasing dilutions.

Measured light scattering signals may originate from other sources than EVs, such as optical and electrical noise and particles in the sheath fluid or buffer. To confirm that background noise sources are negligible, it is important to perform a buffer‐only control in every experiment. The buffer‐only control involves measuring the buffer, which is supposed to be clean and therefore is expected to result in low counts compared to the EV sample.

### 2.3 Fluorescence intensity measurements

Besides light scattering, FCMs can detect EVs labeled with fluorophores, which are used to establish the cellular origin of EVs, or to study the presence of proteins or lipids. Fluorescence occurs after a fluorophore is illuminated by laser light. If the laser wavelength matches the excitation wavelengths of the fluorophore, the fluorophore will go to an excited energy state. After relaxation to its original energy state, the fluorophore emits light at a wavelength longer than the excitation wavelength. More fluorophores bound to an EV yield a higher fluorescence intensity signal.

#### 2.3.1 Fluorophores, antibodies, and generic dyes

FCMs are typically equipped with multiple excitation lasers and spectral filters to enable simultaneous detection of several fluorophores. The lasers and spectral filters of the FCM determine which fluorophores can be used.^12^ Fluorophores that are typically used in EV research are Alexa Fluor dyes, allophycocyanin (APC), fluorescein isothiocyanate (FITC), and phycoerythrin (PE). Alexa Fluor dyes are available in a broad selection of dyes, of which several dyes are excited by wavelengths typically available in FCMs. APC is excited at 633 nm wavelength, and FITC and PE are excited at 488 nm wavelength. The brightness of fluorophores is particularly important for EV detection, because EVs expose a low number of antigens compared to cells. For most FCMs, the fluorescence of part of the labeled EVs will be below the detection limit. Choosing a brighter fluorophore therefore would result in the detection of more EVs.^22^ As a rule of thumb, APC and PE are 5‐ to 10‐fold brighter than FITC.^12^ When combinations of fluorophores are simultaneously used to label EVs, these fluorophores should not overlap in their emission spectrum. Spectral overlap causes multiple fluorophores to be detected by the same detector, resulting in misidentification of EVs.^11^, ^12^ Although for cell analysis methods are available to compensate for spectral overlap, more research is required to develop and validate spectral compensation methods for the analyses of EVs.^10^, ^12^

EVs are typically labeled with fluorophores conjugated to antibodies, which bind to specific antigens exposed by the EVs. As both fluorophores and antibodies consist of mostly proteins, which have a higher RI than the medium, the scatter signal from a stained EVs will likely increase, but we are not aware of any experimental confirmation. The most widely studied body fluid in EV research is plasma.^8^ Plasma contains EVs originating from blood cells, including platelets, erythrocytes, and leukocytes, and the endothelium. The cellular origin of single EVs can be determined by labeling with an antibody that specifically binds to an antigen that is exposed exclusively on EVs originating from a particular cell type. For example, platelets expose the fibrinogen receptor, the integrin α_IIb_β_3_. Antibodies directed against the α_IIb_ and β_3_ subunit of this complex are categorized as cluster of differentiation (CD) 41 and CD61, respectively.^23^, ^24^ Each numbered CD includes all the (clones of an) antibodies that can be bound to a particular surface protein at the surface of EVs, thereby revealing the cellular origin of the EV. Thus, EVs in plasma that bind CD41 and/or CD61 are considered to be “platelet‐derived EVs.” Similarly, antibodies directed against glycophorin A, categorized as CD235a, are considered to identify erythrocyte EVs.^5^, ^6^, ^25^

In addition to antibodies, there are generic dyes of which most stain the phospholipid membrane or primary amines found on the surface of proteins. Thus far, none of these generic dyes label all and exclusively EVs. From five investigated generic dyes, the protein lactadherin, which binds to phosphatidylserine in the membrane of EVs, resulted in the highest sensitivity and specificity to stain EVs >200 nm in diameter.^26^, ^27^ Another protein which binds to phosphatidylserine is annexin V, but annexin V needs calcium ions to bind to phosphatidylserine. Because calcium ions are also a cofactor for coagulation, annexin V is often not recommended to label plasma EVs.^26^, ^28^

#### 2.3.2 From arbitrary units to comparable units of fluorescence

**FIGURE 3:**
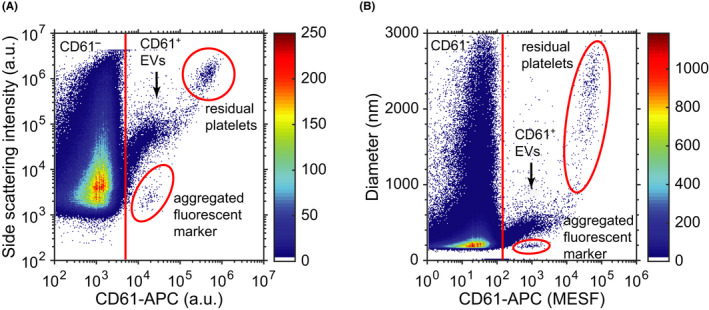
The side scattering intensities and fluorescence intensities of human plasma labeled with cluster of differentiation (CD) 61 conjugated to allophycocyanin (APC) can be used to determine the populations of CD61^+^ extracellular vesicles (EVs), of CD61^‐^ particles, residual platelets, and aggregates originating from the fluorescent reagent. The results are (A) uncalibrated, thus in arbitrary units, and (B) calibrated with the diameter of the EVs in nm and the fluorescence intensity in molecules of equivalent soluble fluorophore (MESF). Without the red outlined regions, uncalibrated results would be difficult to interpret, whereas the calibrated results are insightful and comparable to other data of platelet‐derived EVs

Similar to the scattering intensity, fluorescence intensity is reported in arbitrary units, which are difficult to compare and interpret. Figure 3A shows the side scattering intensities and fluorescence intensities of human plasma labeled with CD61‐APC. At least four populations can be differentiated, but without the red outlined regions, the data are difficult to distinguish. The key to identifying the populations is calibration. As explained earlier, the light scattering signal can be calibrated and related to the diameter of EVs. The fluorescence intensity, in turn, can be calibrated to units of molecules of equivalent soluble fluorophore (MESF). An EV that fluoresces with a given MESF intensity has a similar fluorescence intensity as the number of unlabeled fluorophores present in solution (under the same experimental conditions).^29^, ^30^

Figure 3B shows the same data as in Figure 3A, but on calibrated scales. The side scattering intensity is related to diameter and the fluorescence intensity is expressed in units of MESF. As platelets have typically a diameter between 2 and 3 µm and a fluorescence intensity between 10^4^ and 10^5^ MESF when labeled with CD61‐APC, it becomes immediately clear that the upper right population are residual platelets.^31^ The plot further shows that for this FCM, CD61‐APC^+^ EVs can be differentiated from CD61‐APC^‐^ particles by defining a threshold at 130 MESF. Because the threshold is expressed in MESF, the threshold and fluorescence intensities of EVs can be compared to other FCMs.^10^

#### 2.3.3 Common misinterpretations

There are several misinterpretations that can hinder the detection and proper identification of the fluorescence signal. To validate if the fluorescence signals originate from label‐positive EVs, calibration and controls are required. For example, FCMs that are less sensitive than the FCM in Figure 3 may only detect larger particles than (spherical) EVs, such as residual platelets, pseudopodia, and empty erythrocytes in blood plasma.^5^, ^32^ Because data are in arbitrary units, this misidentification may go unnoticed without calibration.

Another case in which the fluorescence signal does not originate from label‐positive EVs is when the signal originates from autofluorescence. Autofluorescence occurs when biological particles emit fluorescence in absence of fluorophores, giving additional background noise.^11^ The CD61^‐^ band in Figure 3 is caused by both autofluorescence of particles and background noise of the instrument. Unstained samples can be used as a control to determine the autofluorescence of particles and other noise sources contributing to the fluorescence signal.^33^

Additionally, the reagents used for fluorescence labeling may contain aggregated or unbound fluorophores, which can be counted erroneously as a positive event by the FCM. The concentration of aggregates can be reduced by high‐speed centrifugation (eg, 5 minutes at 19 000 *g*). To ensure that the FCM is only measuring labeled EVs, reagents can be added to buffer and measured as a control.^33^ Based on the buffer with reagents control, we identified aggregates in the fluorophores added to the sample measured in Figure 3.

Furthermore, the specificity of the used antibodies has to be confirmed using isotype controls. Isotype controls will determine if antibodies are binding FC receptors on the membrane.^33^

In some assays, it is required to isolate labeled EVs. For example, generic dyes may form micelles themselves, which may be erroneously detected as EVs and therefore must be removed prior to analysis. Because frequently used isolation methods may affect the concentration of labeled EVs, it is good practice to run the buffer‐only, as well as the buffer with reagents by the isolation method, to confirm the absence of positive events resembling EVs. This control was recently introduced as “procedural control”.^33^

An additional pitfall is the limited number of antigens exposed on EVs. The small diameters of EVs result in low numbers of antigens present on their surface compared to cells. It is important to realize that in most assays, a fraction of labeled EVs remains below the detection limit of the flow cytometer.^10^, ^34^ Therefore, the brightness of the fluorophores is important, and bright dyes, such as APC and PE, are preferred.

Also for fluorescence, swarm detection may lead to misinterpretation. When multiple small particles, including aggregated or unbound fluorophores, are simultaneously illuminated, their total (auto)fluorescence might exceed the trigger threshold, thereby contributing to the overall measured fluorescence signal.^35^ Solutions to avoid swarm detection are sample dilution and lowering the sample flow rate.^10^, ^15^ Serial dilution is the control to confirm absence of swarm detection (see also Light scattering).^33^ To reach the eventual goal of reliable measurements of the same properties of EVs between FCMs and laboratories, Table 2 lists the most important pitfalls to evade and techniques to apply.

**TABLE 2:** Pitfalls and solutions of flow cytometry experiments on extracellular vesicles

Pitfall	Solution
Day‐to‐day variation	Apply daily cleaning and regular maintenance and monitor day‐to‐day variation daily using appropriate quality controls for scatter, fluorescence and flow rate.
Dim fluorescence signals	Select the brightest fluorophores and incubate longer during staining.
Event signals originate from noise or other particles than the envisioned EVs	Apply appropriate controls (buffer‐only control, buffer with reagents control, unstained control, isotype control, FMO and single‐stained control, procedural control, serial dilution, detergent treatment) to ensure that events are actual EVs.
FCMs cannot measure all sizes of EVs	Currently, no FCM exist with a dynamic range suitable for measuring all EV sizes. Calibrate scatter and fluorescence of the FCM to know the measurement ranges of your FCM.
Incomparable data between FCMs	Calibrate scatter, fluorescence and flow rate of the FCM daily to generate data in comparable standardized units.
Pre‐analytical variation	Standardize collection, handling and storage of biospecimens
Swarm detection	Dilute the EV‐containing sample, lower the flow rate and validate by titration
Unable to reproduce measurement results	Document each step taken in the pre‐analysis and analysis and calibrate the scatter, fluorescence and flow rate of the FCM^33^
Wrong estimation of EV size	Take the optical configuration of the FCM as well as the difference in RI between EVs and the reference beads into account using Mie theory.

Abbreviations: EVs, extracellular vesicles; FCM, flow cytometer; FMO, fluorescence minus one; RI, refractive index.

### 2.4 Selecting a flow cytometer for EV research

To evaluate the suitability of an FCM to study EVs, we recommend calibrating the scatter and relevant fluorescence detectors. By expressing the sensitivities of detectors in comparable units, sensitivities can be compared to FCMs in the field (eg, Figures 2B and 3B) and to the requirements for future research. Please note that FCM manufacturers typically specify the scatter sensitivities in terms of the smallest detectable diameter of polystyrene beads, which is misleading, because polystyrene beads scatter light substantially more efficient than EVs, as shown in Figure 2B. Furthermore, the flow rate should be stable and measurable. Only after measuring an EV sample on a calibrated FCM, one can decide if the performance of the FCM satisfies the preferred requirements for EV flow cytometry. Although it is beyond the scope of this manuscript to discuss how to set up an FCM, we recommend reading MIFlowCyt‐EV to get an impression of the relevant parameters and assay controls.^33^ The sensitivity of FCMs does not only depend on the brand and type, but also on the state of maintenance and instrument setup.

## 3 GETTING EXTRACELLULAR VESICLES INTO THE CLINIC

Thus far, most if not all studies in which concentration of cell type‐specific EVs were measured by flow cytometry were *single‐center* studies. However, exploration of the real clinical relevance of EVs requires *multicenter* studies, which is feasible only when their results are comparable. In other words, when different instruments and institutes measure the same concentration of cell type‐specific EVs in a given sample. To reach this goal, standardization and insight in the sources of variation are needed, and potential applications will be briefly discussed.

### 3.1 Standardization

There are three sources of variation that affect the outcome of EV measurements. The first source is the sample itself, for example, a body fluid such as blood and urine. The body fluid composition is donor‐dependent and depends for example on age, diurnal rhythm, fasting state, and medication.^33^, ^36^, ^37^ The second source of variation is the pre‐analytical phase, in which the samples are collected, handled, and stored until analysis of EVs. The third source of variation is the analytics, the hardware, and software used to detect EVs and analyze the obtained results.^38^ Because the analytics have been incompletely understood for a long time, monitoring and optimizing the pre‐analytics was difficult. At present, this is rapidly changing, due to international standardization initiatives.

Standardization of the sample itself is possible only to a limited extent. As mentioned before, the composition of a body fluid, including endogenous EVs, will differ per person.^33^ If feasible, it is recommended to collect blood from fasting donors. Fasting donors have a lower concentration of chylomicrons, which makes EV isolation easier and detection faster, because samples can be measured at lower dilutions.^8^, ^39^ Most importantly, samples from, for example, patients and controls should be treated similarly, and pre‐analytical protocols about the collection, handling, and storage of EV‐containing samples can be standardized or should at least be properly documented in such a way that other laboratories can potentially produce similar results.^8^, ^33^, ^40^

Because a large variety of pre‐analytical variables flooded the literature, the EV‐TRACK platform was launched^41^ and various international organizations have produced guidelines for reporting information. The International Society for Extracellular vesicles (ISEV) wrote guidelines about the minimal information for studies of EVs (MISEV).^37^ In addition, there are guidelines about flow cytometry and immunology,^10^ and about collecting, handling, isolating, concentrating, and downstream analysis of EVs by the American Heart Association.^8^ Lastly ISAC (International Society for Advanced Sciences), ISEV and ISTH (International Society for Thrombosis) initiated the EV Flow Cytometry Working Group (evflowcytometry.org), which is committed to improve standardization and educate EV researchers on EV flow cytometry. Last year, the EV Flow cytometry Working Group wrote a reporting framework for EV flow cytometry experiments (MIFlowCyt‐EV).^33^ Only when all details of a flow cytometry experiment are reported, future assays can be standardized.

Standardization of EV detection is essential because it allows, for example, to monitor pre‐analytics. Several interlaboratory comparison studies have been performed, focusing on standardization of the concentration measurements of cell type‐specific (ie, platelet‐derived) EVs in human plasma. In the studies of Lacroix et al (2010) and Robert et al (2009), fluorescent PS reference beads with sizes of 500 nm, 900 nm, and 3 µm, and a plasma sample were distributed.^42^, ^43^ The PS beads were used to set so‐called “microparticle gates” by FSC with the aim to select platelet‐derived EVs. These studies had two shortcomings. Firstly, the difference in RI between the PS beads and EVs was not taken into account. Secondly, the differences in light collection angles of various FCMs were not taken into account. Figure 2, which is based on Mie theory calibration and does consider the RI of EVs and the light collection angles of the FCM, clearly showed that “microparticle gates” select much larger particles than the envisioned EVs. Cointe et al (2017) and Poncelet et al (2016) partly took into account the differences in collection angles, by suggesting different PS bead gates for the FSC and SSC detector. Nevertheless, because FSC and SSC detectors differ between flow cytometers, their approach is not generically applicable and moreover does not provide information about the size of the gated EVs.^44^, ^45^

In 2018, van der Pol et al performed an interlaboratory comparison study wherein the differences in RI between beads and EVs, as well as differences in the optical configuration of the FCMs were taken into acount.^20^ This study showed that in addition to calibrating light scattering, it is equally important to monitor and calibrate the flow rate of the FCM, which for several FCMs were two‐fold higher or lower than the set flow rate. Without the knowledge of the real flow rate it is impossible to draw conclusions about the EV concentration, because the number of EVs that was measured cannot be related to the volume wherein EVs were measured. This study also showed that FCMs of the same brand and type, can differ considerably in sensitivity, which points out that not only calibration is important, but also maintenance and training of the users. Moreover, the study showed that 24% of the tested FCMs have no or only very limited use for EV detection, because 400 nm FITC‐labeled PS beads could not be detected, which corresponds to EVs with an estimated diameter of 700, 1450 or 1790 nm depending on the used FCM.

In 2012, the European Metrology Research Programme (EMRP) project METVES started to develop an infrastructure for robust standardization of EV research. Procedures were developed for collection and handling EV samples from body fluids, and size distributions of commercially available reference beads and EVs were made using both clinical and metrological instruments.^6^ A goal was to achieve traceable measurements of the EV size, meaning that the measured sizes are related to a known reference through a chain of well‐documented calibrations, each contributing to the measurement uncertainty.^46^ However, metrologically traceable measurements of the EV size were not systematically realized due to the polydispersity of EV samples, and lack of suitable reference materials and methods.

Due to the METVES project, it became clear that there are four reasons why currently used reference materials are unsuitable for the calibration of flow rates, fluorescence, and light scattering in EV flow cytometry. First, currently used PS reference beads lack uncertainty statements for the number concentration (flow rate calibration) or MESF (fluorescence calibration). Second, PS beads typically used for flow rate and MESF calibration scatter 1000‐fold more light than EVs, requiring different FCM settings, and are therefore not adapted to EV research. Third, brightness of the dimmest beads used for fluorescence calibration is too high for EV calibrations. Optimally, the brightness should be at least 10‐fold lower.^47^ Fourth, reference beads with an RI in the range of EVs are absent.^47^ The most widely used reference beads are PS and silica beads, which scatter light much more efficiently than EVs, as can be seen in Figure 2. Hence, calibrations based on PS or silica beads and Mie theory require an extrapolation based on the estimated RI of EVs. In reality, however, EVs do not have a single RI, as EVs contain a phospholipid membrane with a high RI and a core with a low but unknown RI. The uncertainty of the RI distribution within EVs therefore contributes to the uncertainty of calibrations based on Mie theory. A promising new reference material are the HOBs, which have a similar structure and RI distribution as EVs. As for today, HOBs are only available in two sizes, but both sizes fall in the RI range of EVs.^16^

The lack of suitable reference materials and procedures for standardizing EV flow cytometry measurements has led to the foundation of the METVES II project (metves.eu), which started in June 2019 (see section Outlook).

### 3.2 Applications

The infrastructure that is being developed will allow the measurements of the concentrations of cell type‐specific EVs in all body fluids. For example, in plasma also EVs originating from a tumour or the placenta may be present.^1^, ^2^ The presence of placenta‐derived EVs is associated with pregnancy and preeclampsia, and therefore EVs have potential as a biomarker. For example, syncytiotrophoblast (STB)‐derived EVs expose the placental alkaline phosphatase, a unique placenta protein, and increased concentrations of STB‐derived EVs have been reported in the plasma of pre‐eclamptic patients compared to normal pregnancy.^1^

Additionally, concentrations of EVs may be measured in, for example, semen, prostate fluid (prostasomes), and follicular fluid. For example, prostasome, that is, EVs released from prostate epithelial cells, may be a potential biomarker for early diagnosis of prostate cancer.^4^

### 3.3 Outlook

The past year, new developments regarding EV FCM and standardization follow‐up quickly. Given the quick generation of often technical knowledge and the interdisciplinary nature of the field, education is important. There are recent initiatives to educate about EV flow cytometry, standardization, and the pre‐analytics. First of all, the EV Flow Cytometry Working Group is working on an educational manuscript on flow cytometry experiments of EVs and organized the EV Flow Series, a series of online seminars on the different subjects within EV flow cytometry, for example about the flow cytometry scatter ratio (Flow‐SR). Flow‐SR is a new method to determine the diameter and RI of EVs, in which the information from two scatter detectors is combined. Flow‐SR can be used for example to discriminate lipoproteins (high RI) from EVs (low RI) without labeling,^7^ although for now the technique can only be applied to particles with a diameter between 200 nm and ~600 nm.

In addition, the METVES II project enables progress in standardization of EV research. In this project, new reference materials with uncertainty statements and properties resembling EVs will be developed to standardize the flow rate, scattering, and fluorescence intensities of EV detection. The reference materials include HOBs, liposomes, and low RI particles. Additionally, a new interlaboratory comparison study will be performed, with the goal to measure the same EV concentrations among participants by (a) full instrument calibration and (b) using a ready‐to‐use and well‐characterized biological test sample. These steps in developing proper reference materials and methods will enable traceable measurements to determine the mean diameter, size distribution width, number concentration, fluorescence intensity, and RI despite the heterogeneity of the EV samples.

Furthermore, a study is being performed to establish reference ranges of EV concentrations in plasma of healthy donors. Nowadays, such reference ranges are lacking, mainly because no FCM is able to detect all EVs. However, with an FCM of which the detectors and the flow rate are calibrated, it becomes feasible to define reference ranges of the EV concentration within known ranges of EV diameter and fluorescence intensity. To make the established reference ranges compliant with clinical standards for reference ranges, blood has been collected from 224 healthy donors. The initial study will not yet deliver metrologically traceable results, although on the long‐term and with the help of the outcomes of METVES II we anticipate that this will become possible. Traceably established reference ranges of EVs in plasma is the next step toward clinical use of EV flow cytometry.

Moving on from improvements in standardization, the hardware of FCMs can still be improved. The design of commercial FMCs precludes the addition of metrological hardware to achieve calibrated, traceable measurements. Therefore, a metrological FCM is being developed as part of the METVES II project. With this metrological FCM, the diameter, RI, and fluorescence intensity of single sub‐micrometer particles can be traceably measured. Additionally, flow sorters are not optimal for EV research, particularly because EVs are co‐sorted with the sheath fluid, thereby risking contamination and requiring a concentration step after sorting.^48^, ^49^ Moreover, only a minor fraction of particles in plasma are the EVs of interest, sorting is a slow process. Traditional sorting hardware therefore require radical changes to make EV sorting more practical.

Lastly, the sensitivity challenge of detecting the smallest EVs in a flow has been recently overcome, but at the expense of throughput. By extending a setup for single‐molecule detection with a flow, Zhu et al^50^ achieved detection of 24 nm silica beads, corresponding to detection of the smallest EVs. Such a nanoparticle FCM, however, does not necessarily preclude the misinterpretations mentioned in the manuscript. For example, with sub‐100 nm particle sensitivity, lipoproteins outnumber EVs in plasma samples and aggregates and micelles originating from staining reagents may become detectable. Moreover, the maximum count rate of the setup of Zhu et al was 200 particles per second, which is low compared to clinical FCMs, which can easily operate beyond detection of 5000 EVs per second. For clinical use, measurements should be fast and reliable, and preferably also the smallest EVs are included in the measurements. Research should be conducted to defeat the tradeoff or determine the optimal tradeoff between speed and sensitivity.

Due to all challenges that EV research has faced over the years, it has become clear that reporting and calibration are key to the realization of standardized and comparable EV flow cytometry experiments. Moreover, using the MIFlowCyt‐EV reporting framework and applying calibrations do avoid the majority of pitfalls that may occur in EV FCM experiments. Although neither clinical FCMs nor currently available reference materials are ideal for EV detection, together they can lead to concordant results and new clinical insights. If EV researchers will start using the MIFlowCyt‐EV framework and calibrations now, we are confident that, upon the arrival of faster and more sensitive FCMs together with improved reference materials and procedures, both comparable FCM measurements and EV‐based biomarkers will become reality.

## CONFLICT OF INTEREST

E. van der Pol is co‐founder and shareholder of Exometry BV.
